# Comparison of medical issues in antenatal and perinatal periods in early youth, adolescent, and young adult mothers in Taiwan: a 10-year nationwide study

**DOI:** 10.1186/1471-2393-14-260

**Published:** 2014-08-04

**Authors:** Chun-Che Huang, Yung-Chieh Lin, Yu-Tung Huang, Kuang-Hua Huang

**Affiliations:** Institute of Health Policy and Management, National Taiwan University, Taipei, Taiwan; Department of Pediatrics, National Cheng Kung University Hospital, & Graduate Institute of Clinical Medicine, College of Medicine, National Cheng Kung University, Tainan, Taiwan; Master Degree Program in Aging and Long-Term Care, College of Nursing, Kaohsiung Medical University, Kaohsiung, Taiwan; The Chronic Diseases and Health Promotion Research Center, Chang Gung University of Science and Technology, Chiayi, Taiwan; Department of Health Service Administration, China Medical University, Taichung, Taiwan

**Keywords:** Adolescents, Maternal age, Delivery, Medical issues

## Abstract

**Background:**

Limited information is available concerning investigating the separate effect of teenage childbirth on medical issues in the antenatal and perinatal periods. Therefore, this study aimed to assess medical problems in antenatal and perinatal periods among early youth, adolescent and young adult mothers in Taiwan.

**Methods:**

This retrospective population-based cohort study was conducted by using data from Taiwan’s National Health Insurance Research Database. A total of 335,590 mothers aged less than 25 years who had singleton births were identified between 2002 and 2011. Univariate and multivariate logistic regression analyses were conducted to estimate unadjusted and adjusted odds ratios (OR) and 95% confidence intervals (CI) of each medical problem category in the antenatal and perinatal periods.

**Results:**

Compared with mothers aged 20–24 years, adolescents (16–19 years) and early youth mothers (≤15 years), particularly those aged 10–15, had a significantly higher risk of intrauterine growth retardation (IUGR, OR = 1.37, 95% CI: 1.00–1.89) and preterm delivery (OR = 2.98, 95% CI: 2.48–3.58) after adjusting for demographic characteristics and clinical factors. Additionally, adolescents mothers were at an increased risk of anemia (OR = 1.32, 95% CI: 1.24–1.40), oligohydramnios (OR = 1.21, 95% CI: 1.12–1.32), failed labor induction (OR = 1.33, 95% CI: 1.24–1.43), and fetal distress (OR = 1.20, 95% CI: 1.14–1.26) after adjustment.

**Conclusions:**

Not all young mothers in our study experienced the same magnitude of increased medical problems in the antenatal and perinatal periods. However, a sufficiently higher probability of having IUGR and preterm delivery was observed among early youth and adolescent mothers.

## Background

Adolescent childbirth, defined as delivery before 20 years of maternal age [1], has a critical impact on individuals, their families, and society as a whole in developed and developing countries. Teenage births comprise approximately one-eighth of all births worldwide [2]. Although recent data have shown a decline in the adolescent birth rate over the past decades, teen pregnancy and birth remain important issues in the field of adolescent health [3–5].

The World Health Organization estimates that female individuals aged 10–19 years account for 23% of the worldwide burden of diseases caused during pregnancy and childbearing [2]. Previous studies also have reported that adolescent women were at higher risk of poor maternal and neonatal outcomes owing to their lack of physical maturity and reproductive development [5, 6]. Despite the ongoing controversy concerning maternal age and its effect on adverse perinatal outcomes [7–10], prior research has shown that adolescent pregnancy is associated with high risk of low birthweight, preterm delivery [7, 11–13], eclampsia [9], anemia [8], premature rupture of membranes (PROM) [13], and fetal death [5, 9] associated with underage pregnancy.

Although adolescent pregnancy has been consistently associated with adverse birth outcomes, inconsistencies in the results may be attributed to different maternal age groups being examined by researchers [7–11, 14–16]. Only a few studies have investigated risk factors and obstetric and perinatal outcomes in young adolescents aged ≤15 years [7, 11, 14]. These studies have also highlighted the importance of age-specific analysis when comparing obstetric and perinatal results between adolescent and young adult mothers.

In addition, specific characteristics of medical institutions and providers may confound the effects of birth outcome. Therefore, age-specific analyses regarding differences in adolescent childbirth factors, especially among younger adolescents, are necessary. This study used 10 years of population-based data to evaluate the medical issues experienced in the antenatal and perinatal periods among early youth, adolescent and young adult mothers in Taiwan.

## Methods

### Data sources

This population-based cohort study were retrieved from Taiwan’s National Health Insurance Research Database (NHIRD) between 2002 and 2011 and included data from inpatient expenditures by admissions, registry for contracted medical facilities, and the registry for medical personnel. The linkage of all datasets for the relevant variables used the scrambled unique personal or medical institutional identification number, which was encrypted by the National Health Insurance Administration (NHIA). Before data release, regulations regarding protection of patient privacy and confidentiality were strictly adhered. The procedure was categorized based on the diagnosis-related group (DRG) of the NHIA; the principal diagnosis code and up to four secondary diagnosis codes for each hospitalization were classified according to the International Classification of Diseases, Ninth Revision, Clinical Modification (ICD-9-CM) coding. In addition, this study was approved by the Research Ethics Committee at China Medical University (approval No. CMU-REC-101-012, Taichung, Taiwan).

### Study population

A total of 335,716 women aged 10–24 years with a live singleton birth between 2002 and 2011, were identified by the DRG and the procedure codes 0373A or 0373C (vaginal delivery) and 0371A or 0373B (cesarean delivery) on the basis of the NHIA’s case-based payment system. We excluded 126 births (0.04%) because the patients’ or their attending physicians’ records were incomplete. Our ultimate sample included 335,590 singleton deliveries.

### Outcome measures

The primary outcome measure was the occurrence of medical issues among singleton births. These medical issues among mothers with singleton births were determined based on prior research and information available in the NHIRD. In this study, we classified the medical issues in the antenatal and perinatal periods.

#### Antenatal medical issue

Numerous studies have documented the medical problems associated with antenatal birth. A pregnant woman is defined as having an adverse problem in the antenatal period [17–19] based on the presence of 1 or more of the following clinical and medical evaluation findings: previous cesarean history (ICD-9-CM code 654.2), prolonged pregnancy (645), preeclampsia (642.4, 642.5, or 642.7), pregnancy-induced hypertension (PIH; 642.0, 642.1, 642.2, 642.3, 642.9, or 760.0), anemia (648.2), intrauterine growth restriction (IUGR; 656.5), oligohydramnios (658.0), and antepartum hemorrhage, placental abruption, placenta previa (641, 762.0, or 762.1).

#### Perinatal medical issue

Several obstetrical studies have reported malpresentation, dystocia, and fetal distress as perinatal medical conditions, which are major indications for cesarean delivery [17, 20, 21]. Mothers were considered to have a perinatal risk factor if they had 1 or more of the following conditions: malpresentation (ICD-9-CM codes 652, 761.7, 763.0, and 763.1), dystocia (653 and 660–662, excluding 661.3), and fetal distress (656.3). Furthermore, we also selected PROM (658.1 or 658.2) and preterm delivery (644), failed induction of labor (659.0 or 659.1), and postpartum hemorrhage (666) as other medical conditions that may be partially attributable to increased adverse medical problems in the perinatal period.

### Main exposure

The main exposure was maternal age of young mothers, classified into 3 groups: early youth mothers (≤15 years), adolescent mothers (16–19 years), and young adult mothers (20–24 years). Women aged 20–24 years, who may have the lowest risk of adverse birth outcomes, were designated as the reference group in all analyses.

### Covariates

We selected covariates based on the available literature as follows: the calendar year of birth, characteristics of the medical institutions (including accreditation level, ownership, and geographic location), attending physicians (including gender and age), and laboring mothers (including birth parity, low-income family status, and delivery mode). Information on institution and provider factors was obtained from the registry for contracted medical facilities and personnel datasets. Medical centers were the hospitals with the best overall evaluation results under Taiwan’s hospital accreditation system, followed by regional hospitals and district hospitals. Hospital ownership was grouped into public, private, and legal foundation-affiliated hospitals. We also categorized the medical institutions into 4 regions (Northern, Central, Southern, and Eastern) on the basis of the geographic location of the institutions in Taiwan. Additionally, the consequences of teenage childbearing were partially due to a complex combination of lower socioeconomic condition [2–4]. We thus used the low-income family status as a proxy for lower socioeconomic status and identified by whether patients were eligible for Taiwan’s National Health Insurance (NHI) premium for low-income households.

### Statistical methods

All analyses were performed using the SAS version 9.2 software packages (SAS Institute, Cary, NC, USA). Distributions of patient, institution and provider characteristics, as well as each medical issue in the antenatal and perinatal periods by maternal age groups were performed. Comparisons of categorical variables were made using the chi-square test, and continuous variables were compared using the Student’s t test. Univariate and multivariate logistic regression models were used to analyze the each medical issue in the antenatal and perinatal periods among adolescent and young adult mothers. Odds ratios (OR) with 95% confidence intervals (CI) were estimated. Statistical significance was set at *p* < 0.05 for each analysis.

## Results

### Demographic and birth related characteristics

In total, 1,199 early youth mothers (aged ≤15 years), 38,716 adolescent mothers (aged 16–19 years), and 295,675 young adult mothers (aged 20–24 years) who had singleton births in Taiwan were identified between 2002 and 2011. Table 1 describes the basic characteristics of the study groups. Crude fertility rates declined during the study period, as Taiwan has experienced a steep fertility decline to 1.15 per woman in 2011. Early youth were more likely to be admitted to non-profit, proprietary, large, and high-level institutions (medical centers or regional hospitals) than the other two groups. In terms of attending physicians, early youth and adolescent mothers were slightly more likely to choose female and older physicians when compared with the young adults. Additionally, early youth and adolescent mothers (25.7% and 22.8%, respectively) were less likely to undergo caesarean delivery when compared with young adult mothers (27.2%). A higher proportion (6.6% for early youth and 2.7% for adolescents) of the mothers came from low-income families when compared with young adult mothers (1.2%).

**Table 1:** **Distribution of singleton sample characteristics by maternal age groups in Taiwan**

Characteristic	Maternal age (years)						p-value
	≤15		16–19		20–24		
	(n = 1,199)		(n = 38,716)		(n = 295,675)		
	no.	(%)	no.	(%)	no.	(%)	
Year of labor							<0.001
2002	219	(18.3)	7,482	(19.3)	48,793	(16.5)	
2003	187	(15.6)	6,114	(15.8)	45,014	(15.2)	
2004	154	(12.8)	5,173	(13.4)	40,903	(13.8)	
2005	114	(9.5)	4,425	(11.4)	35,239	(11.9)	
2006	116	(9.7)	3,462	(8.9)	31,279	(10.6)	
2007	101	(8.4)	3,050	(7.9)	25,635	(8.7)	
2008	84	(7.0)	2,633	(6.8)	21,560	(7.3)	
2009	70	(5.9)	2,301	(5.9)	17,626	(6.0)	
2010	88	(7.3)	2,005	(5.2)	14,553	(4.9)	
2011	66	(5.5)	2,071	(5.4)	15,073	(5.1)	
Institution levels							<0.001
Medical center	178	(14.8)	2,623	(6.8)	23,119	(7.8)	
Regional hospital	322	(26.9)	8,865	(22.9)	67,159	(22.7)	
District hospital	249	(20.8)	10,482	(27.1)	82,286	(27.8)	
Obstetrics/gynecology clinic	450	(37.5)	16,746	(43.2)	123,111	(41.7)	
Institution ownership							<0.001
Public	124	(10.3)	3,138	(8.1)	22,152	(7.5)	
Private for-profit	660	(55.1)	25,773	(66.6)	198,997	(67.3)	
Non-profit proprietary	415	(34.6)	9,805	(25.3)	74,526	(25.2)	
Institution location							<0.001
Northern	514	(42.9)	15,574	(40.2)	122,089	(41.3)	
Central	239	(19.9)	9,056	(23.4)	70,248	(23.7)	
Southern	369	(30.8)	12,068	(31.2)	94,280	(31.9)	
Eastern	77	(6.4)	2,018	(5.2)	9058	(3.1)	
Physician gender							0.011
Female	121	(10.1)	3,364	(8.7)	24,744	(8.4)	
Male	1,078	(89.9)	35,352	(91.3)	270,931	(91.6)	
Physician age, mean (SD)	44.7	(7.1)	44.7	(6.8)	44.4	(6.5)	<0.001
Parity of birth							<0.001
1	1,176	(98.1)	34,435	(88.9)	231,580	(78.3)	
≥2	23	(1.9)	4,281	(11.1)	64,095	(21.7)	
Low-income family status	79	(6.6)	1,061	(2.7)	3,408	(1.2)	<0.001
Delivery mode							<0.001
Caesarean delivery	308	(25.7)	8,812	(22.8)	80,475	(27.2)	
Virginal delivery	891	(74.3)	29,904	(77.2)	215,200	(72.8)	

Values expressed as n (%) or mean (SD).

### Medical issues in the perinatal and antenatal periods

Table 2 summarizes the distribution and univariate analyses of medical issues in the perinatal and antenatal periods, according to maternal age groups. Early youth and adolescent mothers experienced a significantly higher crude rate of IUGR (0.9% and 0.6%, respectively) compared to young adult mothers (0.5%), although they also had a lower crude proportion of cesarean history in the antenatal period. Unfortunately, early youth mothers experienced the highest rate of preterm delivery (<37 weeks gestational age) at 11.3% in the perinatal period, which decreased steadily with maternal age to 5.2% for adolescent mothers and 3.6% for young adult mothers. Additionally, adolescent mothers experienced significantly higher crude rates of anemia (3.8%) and oligohydramnios (0.4%) compared to young adult mothers (3% and 0.3%, respectively). Adolescent mothers also had lower crude proportions of prolonged pregnancy (2.7%) and antepartum hemorrhage and abruptio/previa placenta (0.9%) in the antenatal period, as well as dystocia (8.9%) and malpresentation (4.7%) in the perinatal period.

**Table 2:** **Frequency distribution and univariate analyses of medical issues in antenatal and perinatal periods among singleton births by maternal age groups in Taiwan**

	Maternal age (years)									
	≤15				16–19				20–24	
	(n = 756)				(n = 24,857)				(n = 199,630)	
	n	(%)	OR ^a^ (95% CI)	p-value	n	(%)	OR ^a^ (95% CI)	p-value	n	(%)
**Antenatal medical issue**										
Previous cesarean history	4	(0.3)	0.05 (0.02–0.13)	<0.001	897	(2.3)	0.35 (0.33–0.38)	<0.001	18,663	(6.3)
Prolonged pregnancy	31	(2.6)	0.82 (0.58–1.18)	0.289	1,032	(2.7)	0.85 (0.80–0.91)	<0.001	9,223	(3.1)
Preeclampsia	8	(0.7)	1.03 (0.52–2.08)	0.926	262	(0.7)	1.05 (0.92–1.19)	0.474	1,909	(0.7)
PIH	3	(0.3)	0.76 (0.24–2.35)	0.629	133	(0.3)	1.04 (0.87–1.25)	0.682	978	(0.3)
Anemia	42	(3.5)	1.16 (0.85–1.58)	0.347	1,480	(3.8)	1.27 (1.20–1.34)	<0.001	8,976	(3.0)
IUGR	11	(0.9)	1.93 (1.06–3.50)	0.031	241	(0.6)	1.31 (1.14–1.50)	<0.001	1,412	(0.5)
Oligohydramnios	5	(0.4)	1.31 (0.54–3.16)	0.547	158	(0.4)	1.28 (1.08–1.52)	0.004	942	(0.3)
Antepartum hemorrhage, abruptio/previa placenta	12	(1.0)	0.82 (0.46–1.44)	0.483	350	(0.9)	0.74 (0.66–0.82)	<0.001	3,620	(1.2)
**Perinatal medical issue**										
PROM	47	(3.9)	1.02 (0.77–1.37)	0.873	1,429	(3.7)	0.96 (0.91–1.02)	0.178	11,331	(3.8)
Failed labor induction	5	(0.4)	1.73 (0.72–4.17)	0.225	113	(0.3)	1.21 (0.99–1.47)	0.065	716	(0.2)
Dystocia	108	(9.0)	0.96 (0.79–1.17)	0.683	3,426	(8.9)	0.94 (0.91–0.98)	0.001	27,649	(9.4)
Malpresentation	61	(5.1)	0.83 (0.64–1.08)	0.166	1,801	(4.7)	0.76 (0.72–0.80)	<0.001	17,874	(6.1)
Fetal distress	20	(1.7)	0.80 (0.51–1.24)	0.31	839	(2.2)	1.04 (0.97–1.12)	0.317	6,178	(2.1)
Preterm delivery	136	(11.3)	3.46 (2.89–4.14)	<0.001	1,993	(5.2)	1.47 (1.40–1.54)	<0.001	10,537	(3.6)
Postpartum hemorrhage	7	(0.6)	1.47 (0.70–3.09)	0.313	173	(0.5)	1.12 (0.95–1.31)	0.171	1,182	(0.4)

CI: confidence interval; IUGR: intrauterine growth retardation; OR: odds ratio; PIH: pregnancy-induced hypertenaion; PROM: premature rupture of membrane.

Values expressed as n (%) unless otherwise stated.

^a^Each medical issue in antenatal and perinatal periods among adolescents aged ≤15 and 16–19 years was estimated as compared with adult mothers aged 20–24 years.

After adjusting for demographic characteristics and clinical factors, multivariate analysis revealed that, after adjusting for demographic characteristics and clinical factors, the highest risk of IUGR and preterm delivery (under 37 weeks of gestational age) was noted in early youth mothers (odds ratio [OR] = 1.37, 95% CI: 1.00–1.89 and OR = 2.98, 95% CI: 2.48–3.58, respectively), followed by those aged 16–19 years (OR = 1.30, 95% CI: 1.21–1.40 and OR = 1.58, 95% CI: 1.51–1.66) (Table 3). Moreover, after adjustment, adolescents mothers were also more likely to experience anemia (OR = 1.32, 95% CI: 1.24–1.40), oligohydramnios (OR = 1.21, 95% CI: 1.12–1.32), failed labor induction (OR = 1.33, 95% CI: 1.24–1.43), and fetal distress (OR = 1.20, 95% CI: 1.14–1.26) compared with young adult mothers.

**Table 3:** **Multivariate analyses of medical issues in antenatal and perinatal periods among singleton births by adolescents aged ≤15 and 16–19 years**

	Maternal age (years)			
	≤15		16–19	
	Adjusted OR (95% CI)	p-value	Adjusted OR (95% CI)	p-value
**Antenatal medical issue** ^**a**^				
Previous cesarean history	0.09 (0.04–0.20)	<0.001	0.43 (0.41–0.46)	<0.001
Prolonged pregnancy	0.54 (0.39–0.77)	<0.001	0.84 (0.79–0.90)	<0.001
Preeclampsia	0.69 (0.47–1.02)	0.063	0.98 (0.91–1.05)	0.501
PIH	0.52 (0.31–0.88)	0.014	0.93 (0.86–1.02)	0.11
Anemia	1.00 (0.73–1.38)	0.991	1.32 (1.24–1.40)	<0.001
IUGR	1.37 (1.00–1.89)	0.042	1.30 (1.21–1.40)	<0.001
Oligohydramnios	0.82 (0.54–1.25)	0.349	1.21 (1.12–1.32)	<0.001
Antepartum hemorrhage, abruptio/previa placenta	0.77 (0.53–1.12)	0.175	0.76 (0.70–0.82)	<0.001
**Perinatal medical issue** ^**b**^				
Premature ROM	0.80 (0.60–1.07)	0.131	0.97 (0.92–1.03)	0.294
Failed labor induction	1.33 (0.97–1.83)	0.075	1.33 (1.24–1.43)	<0.001
Dystocia	0.82 (0.67–1.01)	0.066	0.86 (0.83–0.90)	<0.001
Malpresentation	0.94 (0.75–1.17)	0.57	0.92 (0.88–0.97)	<0.001
Fetal distress	0.78 (0.58–1.05)	0.102	1.20 (1.14–1.26)	<0.001
Preterm delivery	2.98 (2.48–3.58)	<0.001	1.58 (1.51–1.66)	<0.001
Postpartum hemorrhage	1.03 (0.74–1.43)	0.856	1.00 (0.94–1.08)	0.917

CI: confidence interval; IUGR: intrauterine growth retardation; OR: odds ratio; PIH: pregnancy-induced hypertenaion; PROM: premature rupture of membrane.

^a^Adjusted for the year of labor, institution and physician characteristics, parity of birth, and low-income family status, as compared with mothers aged 20–24 years.

^b^Adjusted for the year of labor, institution and physician characteristics, parity of birth, low-income family status, and delivery mode, as compared with adult mothers aged 20–24 years.

## Discussion

This nationwide population-based study in Taiwan revealed that adolescent mothers, particularly those aged ≤15 years, suffered a higher probability of having IUGR and preterm delivery (under 37 weeks of gestational age) compared with mothers aged 20–24 years during the 10-year observation period. Our finding is consistent with previous studies [7, 10, 11, 14, 22] suggesting that young maternal age is associated to an increased risk of preterm delivery, even after controlling for covariates. This higher risk among adolescents was partially explained by other accompanying medical problems, poor maternal diet and nutrient intakes, and inadequate prenatal care [10, 12]. However, other studies reported little or no significant difference between adolescence and preterm delivery [13, 15, 16, 21], although these were not mutually exclusive and could be attributed to a combination of these factors.

Prior research demonstrated that teenagers also had other complicated pregnancies, with higher rates of anemia [5, 8, 14], oligohydramnios, and PIH [14], all of which were associated with increased probability of preterm delivery. Therefore, this proportion of preterm delivery was sufficiently greater for early adolescent mothers and had an important influence on their birth-related morbidity and neonatal mortality [5, 10]. In our study, the higher rates of anemia, oligohydramnios, failed labor induction, and fetal distress were also observed in adolescent mothers (aged 16–19 years). This finding was partially consistent with that of earlier research that indicated that pregnant teenagers more frequently experienced maternal anemia, oligohydramnios, and fetal distress [8]. This consequence may be associated with maternal nutrient deficiencies and accompanying medical problems in the antenatal and perinatal periods among adolescent mothers [23, 24]. Additionally, women with a diagnosis of IUGR and those who delivered low birth-weight infants had significantly less weight gain during pregnancy [12, 14]. However, other studies reported that these adverse outcomes were much higher among pregnant adolescents 15 years or younger [5, 14]. This change in rate was insignificant may be due to the small number of observed cases.

On the contrary, our results revealed that adolescent mothers had lower proportion of previous cesarean history, prolonged pregnancy, antepartum hemorrhage, abruptio/previa placenta, dystocia, and malpresentation compared with young adult mothers. This was partially consistent with a nationwide study in Taiwan, indicating lower rates of breech presentation and dystocia during delivery among women aged < 20 years than among other older age groups [21]. Dystocia and malpresentation were the main indications for emergency cesarean delivery [20, 21]. Additionally, women with cesarean history or antepartum hemorrhage were more likely to undergo cesarean delivery [21]. Consistent with prior research [10, 12–15, 21], we also noted that cesarean delivery rate was lower in the adolescent mothers than in their older counterparts.

However, among the younger age groups in this study, the number of observed cases was relative small size to show the differences in birth-related outcomes between adolescents and young adult mothers. Although prior research suggested that teenage pregnancy and childbearing at risk for life-threatening conditions had an important impact on maternal and neonatal morbidity, and mortality worldwide [2, 5], not all adolescent mothers in this study experienced the same magnitude of medical issues in the antenatal and perinatal periods.

The strengths of this study, including the large sample size and robust adjustment for comprehensive characteristics of laboring women, institutions, and providers, allowed us to obtain an accurate estimate of the independent contribution of the adolescent mothers with singleton births to the identification of medical problems in the antenatal and perinatal periods. While the multifactorial causes of adverse birth outcomes were complex, the present study highlights several areas where future research might illuminate some of the findings.

Several limitations to this study should be considered. First, adjustment for gestational age at delivery or fetal birth weight was not performed. However, circumstantial evidence suggests that this may not be a major issue [21]. We used diagnosis codes to record maturity before and after childbirth, which included most clinically significant deviations from the optimum gestational age. Despite these safeguards, some degree of coding error in administrative data is still possible. Second, information about other unmeasured factors that may influence adverse medical problems, such as prenatal care, body mass index, or maternal lifestyle habits, including smoking or drinking [10, 12], were not available. Owing to data limitation, maternal education level was also not assessed. We used the low-income family status as a marker of socioeconomic status in our analyses.

## Conclusion

This study provided sufficient evidence to detect differences in the magnitude of medical issues in the antenatal and perinatal periods of adolescent mothers in Taiwan. The results indicate that adolescent mothers aged 16–19 had a higher risk of medical problems, but the risk did not appear to increase significantly among those aged ≤15 years. In addition, as adolescent pregnancy and childbirth can affect maternal and neonatal health, as well as familial and societal wellbeing, we suggest that proactive efforts are necessary to prevent adolescent pregnancies. When they do occur, special care must be taken to reduce the incidence of medical problems in the antenatal and perinatal periods.

## Abbreviations

CI: Confidence interval
DRG: Diagnosis-related group
ICD-9-CM: International Classification of Diseases, Ninth Revision, Clinical Modification
IUGR: Intrauterine growth restriction
NHI: National Health Insurance
NHIA: National Health Insurance Administration
NHIRD: National Health Insurance Research Database
OR: Odds ratio
PIH: Pregnancy-induced hypertension
PROM: Premature rupture of membrane
WHO: World Health Organization.
